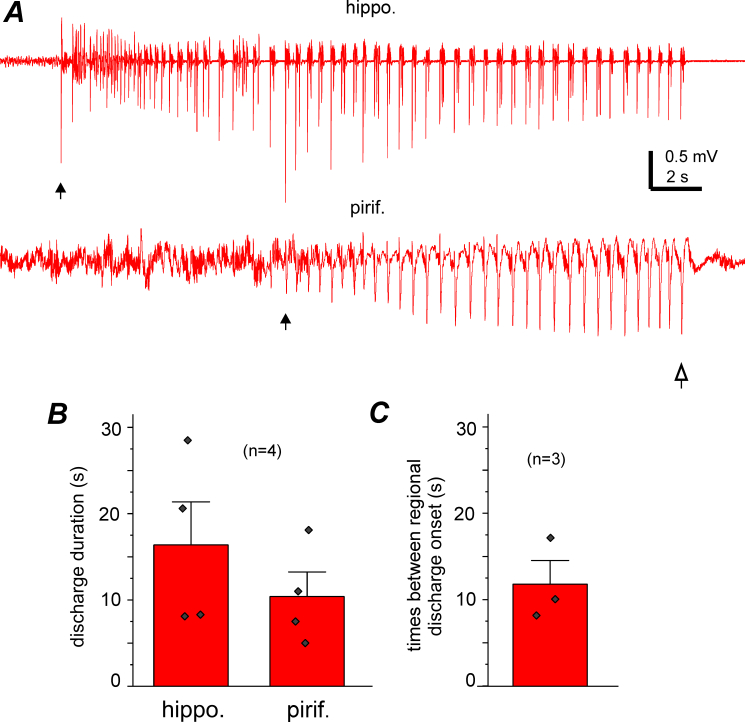# Sex influences on hippocampal kindling-induced seizures in middle-aged mice

**DOI:** 10.1016/j.heliyon.2024.e40294

**Published:** 2024-11-14

**Authors:** Hongmei Song, Yapeng Liu, Yuqing Sun, Bryan Mah, Yang Bai, Liang Zhang

**Affiliations:** a Departments of Neurosurgery and Neuro-Oncology, The First Hospital of Jilin University, Changchun, Jilin, China; b Department of Cardiology, Qilu Hospital, Cheeloo College of Medicine, Shandong University, Jinan, China; c Krembil Research Institute, University Health Network, Toronto, Ontario, Canada; d Division of Neurology, Department of Medicine, University of Toronto, Toronto, Ontario, Canada

**Keywords:** C57 black mice, Convulsion, EEG, Epilepsy, Female, Interictal spikes, Local filed potentials, Spontaneous seizures, Theta rhythm

## Abstract

Epilepsy is a chronic neurological disorder, and its prevalence presents a bimodal distribution with high incidences in children and older adults. The incidence of epilepsy does not generally differ between men and women; however, whether this holds true for new-onset epilepsy in older adults is unclear. While studies in animal models of epilepsy may help explore the biological mechanisms relevant to the influences of sex on epileptogenesis, relatively little information is available regarding sex differences in the genesis of epileptic seizures in middle-aged animals. In this study, we addressed this knowledge gap using a mouse model of extended hippocampal kindling. C57 black mice aged between the ages of 12 and 13 months underwent hippocampal kindling as this age roughly corresponds to middle age in humans (∼50 years). Relative to male mice, female mice showed faster-progressing and more severe evoked seizures, a higher tendency to experience spontaneous seizures in the early stage of extended kindling, less frequent expression of hippocampal interictal spikes, and insignificant decreases in hippocampal theta rhythm. Collectively, these results demonstrated the existence of sex-specific differences in hippocampal kindling-induced seizures and suggested that middle-aged female mice have greater but variable susceptibility to hippocampal kindling-induced epileptogenesis compared with male mice of similar age.

## 1 Introduction

Epilepsy is defined as at least two unprovoked seizures occurring more than 24 h apart, one unprovoked seizure with a high risk of recurrence, or the diagnosis of an epilepsy syndrome [1]. Temporal lobe epilepsy (TLE) is the most common form of the disease seen in adult and aging populations and has highly diverse etiological factors, clinical-electroencephalographic (EEG) manifestations, and brain pathologies [2]. The incidence of epilepsy generally does not differ between men and women; however, whether there are sex-specific differences in new-onset epilepsy in adult/aging populations is unclear [3]. Understanding the latter is important because the incidence of epilepsy increases steadily in adults after the age of 50 years [4]. However, clinical investigations relating to the influence of sex on epilepsy susceptibility are complicated by the existence of complex interactions between biological and socio-economic factors. Animal models of epilepsy may help shed light on the relevant biological mechanisms.

An increasing number of studies have examined the influence of sex on the genesis of epileptic seizures in a variety of animal models [5]. Particularly pertinent to our present work are studies that modeled TLE in C57 black mice, an inbred strain widely used in neurobiology- and epilepsy-related research [6,7]. Similarities and differences in epileptic seizure genesis between female and male C57 mice have been observed in kainic acid [[8], [9], [10], [11]], pilocarpine [12,13] or kindling [[13], [14], [15], [16]] models. The variable influence of sex on seizure outcomes observed from C57 black mice and other animal species [5] is not unexpected as animal models of TLE differ in seizure induction approaches and seizure genesis mechanisms. However, except for two reports [8,16], which included some aging/aged (≥14-months-old) C57 black mice, all studies on TLE models to date have employed young or adult animals, and relatively little is known regarding the influence of sex on epileptic seizure genesis in middle-aged or older animals. Accordingly, in this study, we aimed to provide novel information in this respect using a mouse model of extended hippocampal kindling [[17], [18], [19]]. Specifically, we conducted hippocampal kindling in female and male C57 mice between the ages of 12 and 13 months and explored influences of sex on genesis of evoked and spontaneous seizures in these middle-aged mice.

## 2 Methods

### 2.1 Animals

C57 black mice (C57BL/6J; females and males of 6–8 months-old) were obtained from Charles River Laboratory (Saint-Constant, Quebec, Canada). These mice belonged to different cohorts of retired breeders, not littlemates. Mice were housed in a local vivarium for up to 6 months before the experimentations described below. The vivarium was maintained at room temperature between 22 and 23 °C with a 12-h light on/off cycle (lights on starting at 6:00 a.m.). C57 black mice have a maximum lifespan of ≥30 months, and mice of 11–13 months-old may roughly correspond to human’s ages around 50 years [20]. We therefore choose to kindle C57 mice of 12–13 months-old. All experimentations described below were reviewed and approved by the Animal Care Committee of the University Health Network in accordance with the Guidelines of the Canadian Council on Animal Care.

A previous study of our lab has observed age-related differences in hippocampal kindling seizures between young and aging male C57 black mice (2–3 and 18-22 months-old; 15–18 mice per age group, [21]). Based on this study, we aimed to compare kindling seizures between middle-aged female and male mice of similar sample sizes. However, kindling experiments were unsuccessful in some mice (6 females and 5 males) due to a loss or malfunction of implanted electrodes, and data presented below were collected from 10 female and 12 male mice that experienced ≥50 hippocampal kindling stimulations.

### 2.2 Electrode implantation

The procedures for electrode construction and implantation were similar as previously described [22,23]. All electrodes were made of polyamide-insulated stainless-steel wires (110 μm outer diameter; Plastics One, Ranoake, Virginia, USA). Each mouse was implanted with two pairs of twisted wire bipolar electrodes. The first electrode pair was positioned to the hippocampal CA3 region (bregma −2.5 mm, lateral 3.0 mm, and depth 3.0 mm; [24]) to deliver kindling stimulation and to record local field potentials (LFPs). The second electrode pair was to target non-stimulated ipsilateral piriform cortex (bregma: +0.5 mm, lateral: 3.0 mm, and depth: 5.0 mm) or parietal cortex (bregma: −0.5 mm, lateral: 2.0 mm, and depth: 0.5 mm) [[17], [18], [19]]. A mono-polar reference electrode was positioned at a frontal lob area (bregma +1.0 mm, lateral 1.0 mm, and depth 0.5 mm).

### 2.3 Hippocampal kindling

Hippocampal kindling was conducted using a standard protocol [[17], [18], [19],^21^,[25], [26], [27]]. Constant current pulses (monophasic square waveforms, pulse duration of 0.5 ms and current intensities of 10–100 μA) were generated by a Grass stimulator (model S88, Grass Medical Instruments, Warwick, RI, USA), delivered through a Grass photoelectric isolation unit (model PSIU6) and applied to the twisted wire bipolar electrode. Initially, an ascending series was performed to determine AD thresholds for individual mice. Subsequent kindling stimulations were applied at 25 % above the AD threshold. Each mouse was placed in a large bowl-shaped glass container or a large glass beaker for kindling stimulation and LFP-video monitoring. Kindling stimulations were applied twice daily of ≥5 h apart. Each mouse was considered to reach a kindled state if it showed three consecutively evoked stage 5 motor seizures [21,26,27]. A protocol of extended hippocampal kindling (≥50 stimulations) was used to induce spontaneous seizures [[17], [18], [19]].

### 2.4 LFP recordings

Differential recordings *via* the twisted wire bipolar electrodes and microelectrode AC amplifiers (model 1800 or 3000, AM Systems; Sequim, Washington, USA) were used to monitor LFP signals [[17], [18], [19],[21], [22], [23]]. The amplifiers were set with an input frequency band of 0.1–1000 Hz and amplification gain of 1000 or 3000. Amplifier output signals were digitized at 5000 or 10,000 Hz (Digidata 1440A or 1550, Molecular Devices; Sunnyvale, California, USA). Data acquisition, storage and analyses were done using pClamp software (Version 10; Molecular Devices). The mode 3000 amplifier was used to capture evoked AD from the stimulated hippocampus *via* TTL-gated switch between recording and stimulating modes, and its input frequency band was set in the range of 1/10–1000 Hz to minimize stimulating/switching related artefacts. As such, evoked ADs recorded from the stimulated hippocampal area were more variable in waveform and/or magnitude relative to those recorded from non-stimulated piriform or parietal cortical areas (Supplemental Fig. 1).

### 2.5 Continuous LFP-video monitoring

The protocol and apparatus for continuous 24-h LFP-video monitoring were detailed previously [23]. Each mouse was placed in a modified cage with food and water *ad libitum*. Dim or red lighting was used to facilitate webcam monitoring during the light-off period. A cursor auto-click program (Mini Mouse Macro program; http://www.turnssoft.com/mini-mouse-macro.html) was used to operate LFP and video recordings and save data every 2 h. LFP and video data were collected continuously except a period of about 30 min per day for the purpose of animal care. Individual mice were monitored for 24–48 h before kindling (baseline) and following different numbers of hippocampal stimulations. If ≥ 2 spontaneous seizures were observed over a 24-h period, kindling stimulation was terminated and LFP-video monitoring was continued for up to 4 consecutive days to assess daily incidences of spontaneous seizures. Previous studies of our lab have shown that most male C57 black mice exhibited spontaneous seizures following ≥100 hippocampal kindling stimulations [18] and that about 50 % of mice in a model of neurofibromatosis type 1 exhibited spontaneous seizures following 40 kindling stimulations [28]. Based on these results, LFP-video monitoring was performed after 50, 80, 100 and ≥ 120 stimulations in the present experiments. We used this protocol to detect spontaneous seizures that may occur in the early-intermediate part of extended hippocampal kindling while accommodating continuous LFP-video monitoring in individual mice.

### 2.6 Data analysis

Evoked AD and spontaneous discharges were recognized by repetitive spike waveforms that had amplitudes of ≥2 times of background signals and durations of ≥5 s [18,21,22]. Both evoked AD and spontaneous discharges often ended with a sudden cessation of spike waveforms and a subsequent signal suppression period lasting several seconds. The duration of evoked AD was measured from the end of applied stimulation pulses to the spike waveform cessation. As nearly all spontaneous discharge presented low voltage signals at onset [18], discharge durations were measured from the low voltage signals to the spike waveform cessation.

Evoked and spontaneous motor seizures were analyzed by video readings. Motor seizure severities were scored according to the Racine stage [29] modified for mice [13,26]. Briefly, stage 0 – no response; stage 1 – behavioral arrest; stage 2 – chewing and head nodding; stage 3 – unilateral and/or bilateral forelimb clonus; stage 4 − rearing; stage 5 – falling.

Hippocampal interictal spikes (ISs) and IS-associated with fast ripples (FRs) were measured from 10-min data segments that were sampled in periods where mice were asleep or in stable immobility. A threshold-based detection program (pClamp/Clampfit) was used to automatically detect ISs or FRs [17,19,30]. The detection parameters were set to recognize ISs that had amplitudes of ≥6 standard deviation of mean background signals and durations of ≤200 ms. To assess FRs, original IS signals were treated with a band-passing filter (Bessel, 250–500 Hz) for FR detections. A FR event was detected if it comprised ≥4 consecutive oscillatory events that had amplitudes of ≥3 standard deviation of filtered mean background signals and inter-event intervals of ≤4 ms [31,32]. All detected events were visually inspected, and false events were rejected.

To assess hippocampal theta rhythms, 3–6 data segments with each 0.5–3 min long and a cumulative length of 5–12 min were sampled from individual mice before kindling and following 50 and 80 stimulations. Spectral plots were generated from individual segments (rectangular function, spectral resolution of 0.3 Hz, and averaged with 50 % window overlap, pClamp/Clamfit) and then averaged for measurements of theta peak frequencies and corresponding powers [19].

### 2.7 Statistical tests

Origin (2023B version, Northampton, MA, USA) or GraphPad Prism (version 10.2, Boston, MA, USA) software was used for statistical tests. Data were presented as the mean and the standard error (SE) of the mean throughout the text and figures. Statistical significance was set at *p* < 0.05. Data normality was assessed using the Shapiro-Wilk test. For assessing group differences, the student’s t-test or one-way ANOVA followed by a post-hoc test was used for normally distributed data, and the Mann–Whitney U or Kruskal–Wallis test followed by Dunn’s post-hoc test was employed for non-normally distributed data. Chi-square or Fisher’s exact test was used for rate comparison. Measures of AD thresholds were analyzed using a two-way ANOVA.

## 3 Results

The data presented below were collected from 10 female and 12 male mice that received ≥50 hippocampal kindling stimulations.

### 3.1 Body weights and AD thresholds

The body weights of female and male mice before hippocampal kindling (baseline) were 24.2 ± 0.71 and 29.4 ± 0.99 g, respectively, and were significantly lower in females than in males (Mann–Whitney *U* test, *u* = 7.5, *z* = −3.21, *p* = 0.0014). Measured weekly for 8 weeks following hippocampal kindling, the body weights of female and male mice were in the ranges of 27.5 ± 1.26 g to 28.4 ± 1.30 g and 28.6 ± 0.83 g to 29.9 ± 0.97 g, respectively (Fig. 1A). There were no significant sex-related differences in any of these weekly measures (two-sample *t*-test or Mann–Whitney *U* test, *p* ≥ 0.09). A progressive gain in body weight was not evident in kindled female and male mice as differences among weekly measures of body weights in each group were not significant (Kruskal–Wallis test, Chi-square = 9.98 or 2.03, *df* = 8, *p* = 0.266 or 0.980). The lack of evident body weight gain is dissimilar to that previously observed in male or female rats following amygdala kindling [[33], [34], [35], [36]]. This discrepancy may be due to differences in experimental variables, including kindling sites and animal species used. While examinations of non-kindle controls are needed to verify the absence of effects of hippocampal kindling on body weight in our model, our present observations are in keeping with the mean body weights of age-matched naïve C57 black mice (The Jackson Laboratory; https://www.jax.org/jax-mice-and-services/strain-data-sheet-pages/body-weight-chart-000664). The lack of an apparent loss of body weight in the kindled mice suggested that extended hippocampal kindling did not cause substantial physical deterioration in middle-aged mice.

**Fig. 1:**
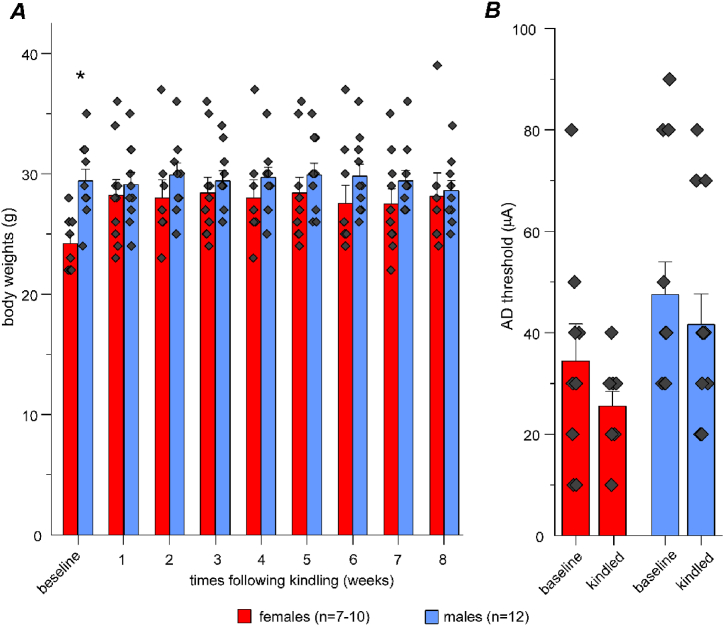
Measures of body weights and after-discharge (AD) thresholds. Data obtained from 7 to 10 female and 11–12 male mice. Here and in subsequent figures, bar graphs presented mean and SE with overlapped individual data points. **A**, body weight measures obtained before (baseline) and weekly following hippocampal kindling. Baseline body weights were significantly lower in female mice relative to male mice (Mann–Whitney *U* test, *u* = 7.5, *z* = −3.21, *p* = 0.0014). There was no significant difference among measures within each group (Kruskal–Wallis test, Chi-square = 9.98 or 2.03, *df* = 8, *p* = 0.266 or 0.980). **B**, AD thresholds assessed before kindling (baseline) and after reaching the kindled state. Original data were transformed using Y = log(Y) function and then analyzed by a two-way ANOVA. There was no significant interaction between the effects of sex and kindling (*f* (1, 38) = 0.002, *p* = 0.966). Sex (*f* (1, 38) = 7.488, *p* = 0.0094), but not kindling (*f* (1, 38) = 0.9949, *p* = 0.3249), had significant effects on AD thresholds.

In our experiments, hippocampal stimulations were applied to individual mice at 25 % above their initial AD thresholds [17, 18, 21, 26, 27]. The AD thresholds measured from individual mice before kindling and after reaching a kindled state (three consecutively evoked stage 5 motor seizures) are presented in Fig. 1B. Original measures were transformed using Y = log(Y) function and then analyzed using a two-way ANOVA to explore the effects of sex and kindling on AD threshold changes. There was no significant interaction between the effects of sex and kindling (*f* (1, 38) = 0.002, *p* = 0.966). Sex (*f* (1, 38) = 7.488, *p* = 0.0094), but not kindling (*f* (1, 38) = 0.9949, *p* = 0.3249), had significant effects on AD thresholds. While this analysis suggested sex influences on AD thresholds in our model, the sex differences in original AD threshold measures were relatively small. We therefore speculate that the strength of hippocampal kindling stimulation might not be a major determining factor for the sex-related differences in kindling seizure outcomes presented below.

#### 3.1.1 Evoked motor seizures

**Fig. 2:**
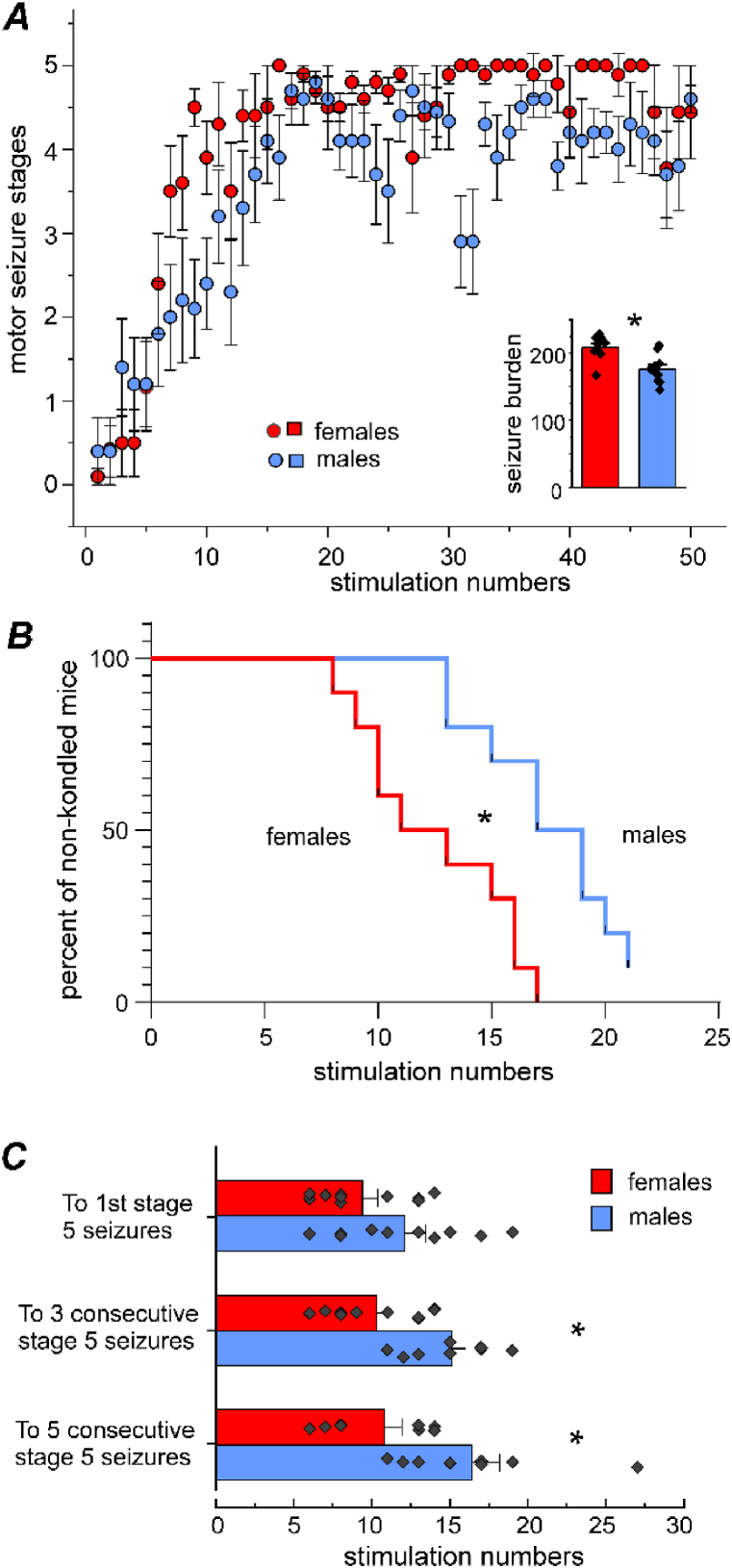
Measurements of evoked motor seizures. **A**, motor seizure stages (y) plotted against numbers of hippocampal stimulations (x). Data (mean and SE) obtained from 10 female and 12 male mice. Insert, seizure burdens (seizure stages x numbers of evoked seizures) imposed by 50 stimulations were greater in female than in male mice (mean ± SE with individual data points; ∗, two-sample *t*-test, *t* = 3.4, *df* = 16, *p* = 0.0036). **B**, Kaplan-Meier curves of kindling seizure progression. Female mice were kindled faster relative to male mice (∗. Long-rank test, Chi-square = 9.427, *df* = 1, *p* = 0.0021). **C**, numbers of hippocampal stimulations needed to elicit stage 5 motor seizures (mean ± SE with individual data points). Female mice required fewer stimulations to exhibit three and five consecutive stage 5 seizures as compared to male mice (∗, two-sample *t*-test, *t* = −3.63598, *df* = 17, *p* = 0.00204; Mann–Whitney *U* test, *u* = 14, *z* = −2.08163, *p* = 0.03738).

Examples of stage 5 motor seizures evoked in female and male mice are presented in Supplementary videos 1–2. To reveal the progression of evoked motor seizures, we plotted the mean seizure stage against the number of hippocampal stimulations that elicited these seizures (Fig. 2A). In response to the first 15 hippocampal stimulations, the motor seizure stages gradually increased as the number of applied stimulations increased in both female and male mice; however, the increases were noticeably more rapid in the former. We used the Kaplan-Meier curve to compare sex-specific differences in kindling seizure progression (Fig. 2B). The proportions of mice that reached the kindled data were significantly different between the female and male groups (Long-rank test, Chi square = 9.427, *df* = 1, *p* = 0.0021), with female mice being kindled faster than male mice.

We also quantified the number of kindling stimulations needed to elicit stage 5 motor seizures in individual mice and pooled them according to sex. As presented in Fig. 2C, it took 9.4 ± 0.97 and 12.1 ± 1.34 stimulations to elicit the first stage 5 seizure, 10.3 ± 0.97 and 15.1 ± 0.890 stimulations to elicit three consecutive stage 5 seizures, and 10.8 ± 1.14 and 16.4 ± 1.8 stimulations to elicit five consecutive stage 5 seizures in female and male mice, respectively. There were significant sex-related differences in the latter two measures (two-sample *t*-test: *t* = 3.64, *df* = 17, *p* = 0.002; Mann–Whitney *U* test: *u* = 14, *z* = −2.08, *p* = 0.037), with female mice requiring fewer stimulations to exhibit consecutive stage 5 seizures.

In response to 16–50 hippocampal stimulations, stage 5 motor seizures were more frequently observed in female mice than in male mice (Fig. 2A). The mean seizure stages evoked by 16–50 stimulations were significantly greater in female than in male mice (4.73 ± 0.05 vs. 4.16 ± 0.07; Mann–Whitney *U* test: *n* = 331 and 348, *u* = 77211, *z* = 8.70, *p* < 0.0001). We also assessed total seizure burdens (seizure stages x numbers of evoked seizures) imposed by 50 hippocampal stimulations in individual mice (Fig. 2A, insert). When pooled them according to sex, the total seizure burden was significantly greater in female mice relative to male mice (208.4 ± 6.2 *vs.* 175.4 ± 7.4; two-sample *t*-test, *t* = 3.40, *df* = 16, *p* = 0.0036). Collectively, these observations indicated that hippocampal kindling induced faster-progressing and more severe motor seizures in female mice than in male mice.

#### 3.1.2 Evoked hippocampal AD

**Fig. 3:**
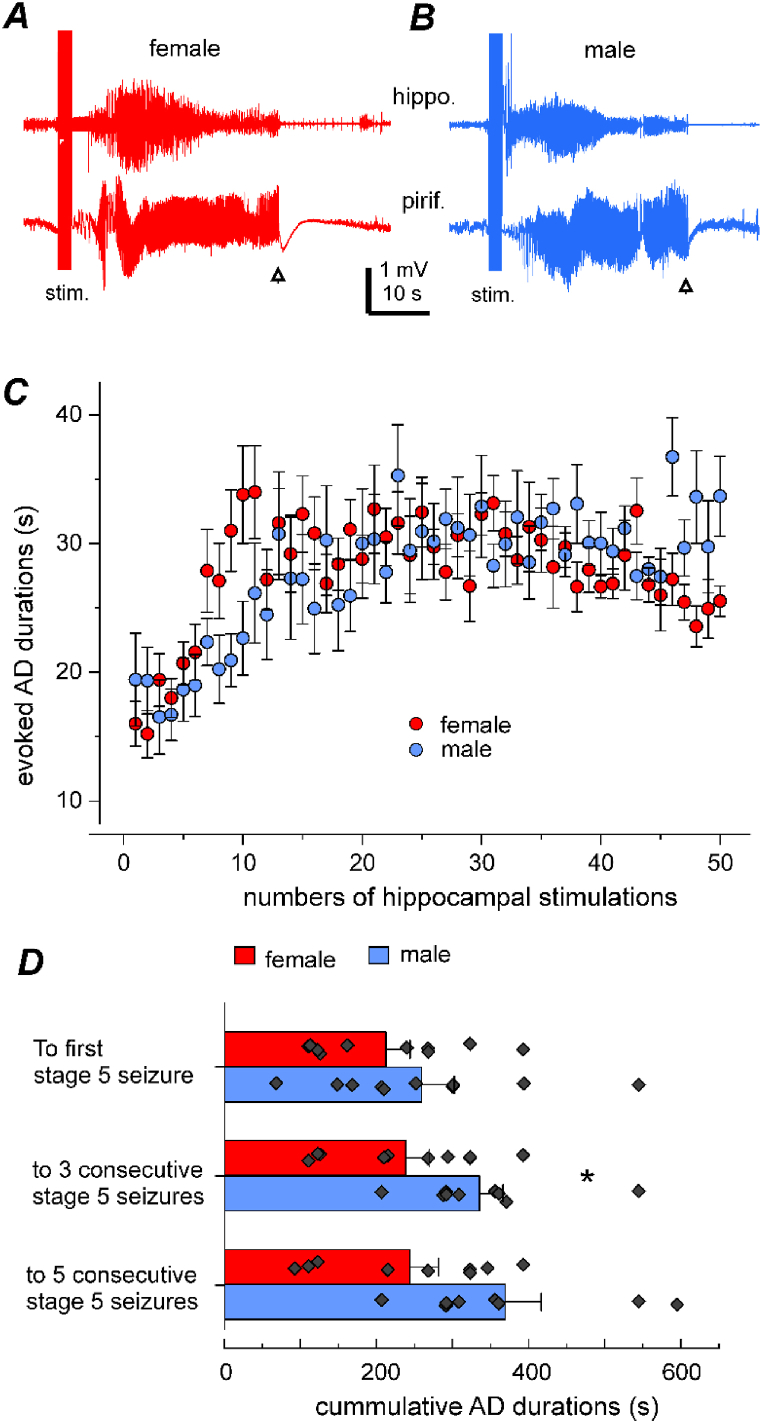
Measurements of evoke after-discharges (AD). **A-B**, examples of evoked AD recorded from the stimulated (stim.) hippocampus (hippo.) and ipsilateral piriform (pirif.) cortex. Corresponding stage 5 motor seizures presented in supplementary videos 1-2 respectively. AD cessation indicated by open arrows. Hippocampal signals recorded in a frequency band of 10–1000 Hz to minimize stimulation artefacts. **C**, AD durations (y, mean ± SE) plotted against numbers of hippocampal stimulations (x). Data obtained from 10 female and 12 male mice. **D**, AD durations (mean ± SE with individual data points) accumulated to the expressions of stage 5 seizures. Females needed shorter AD durations to exhibit three consecutive stage 5 seizures (∗, two-sample *t*-test: *t* = −2.2073, *df* = 17, *p* = 0.041).

In our experiments, LFP signals were simultaneously recorded from the stimulated hippocampal and unstimulated piriform or parietal cortical areas. Examples of evoked AD collected from a female mouse and a male mouse are presented in Fig. 3A and B. Stage 5 motor seizures associated with these discharges are shown in Supplementary videos 1–2. To reveal the progression of local epileptic activity, we measured the durations of hippocampal ADs and plotted the mean AD durations against the number of hippocampal stimulations that evoked these ADs (Fig. 3C). In response to the first 15 kindling stimulations, both females and males showed gradual increases in AD durations as the number of stimulations increased. In response to subsequent 16–50 stimulations, the mean durations of the evoked AD were comparable between female and male mice (28.7 ± 0.38 and 30.4 ± 0.51 s, respectively; Mann–Whitney *U* test: *u* = 50566.5, *z* = −1.33, *p* = 0.184).

We also measured the cumulative AD durations until stage 5 motor seizures as per previous studies [37,38]. The cumulative durations of hippocampal ADs were 211.7 ± 31.61 and 258.4 ± 42.83 s to the first stage 5 seizures, 237.6 ± 30.86 and 334.5 ± 31.05 s to three consecutive stage 5 seizures, and 242.9 ± 47.06 and 368.4 ± 47.06 s to five consecutive stage 5 seizures for female and male mice, respectively (Fig. 3D). Although the difference was significant only for the second measure (two-sample *t*-test: *t* = −2.2073, *df* = 17, *p* = 0.041), overall, female mice required shorter cumulative hippocampal ADs to experience stage 5 motor seizures. These sex-specific differences observed in evoked AD also suggested that evoked seizures progressed faster in female mice than in male mice.

#### 3.1.3 Spontaneous seizures

We performed continuous LFP-video monitoring to detect spontaneous seizures and other relevant activities. Individual mice were monitored for 24–48 h before hippocampal kindling (baseline) and after 50, 80, 100, and 120 stimulations. If ≥ 2 spontaneous seizures were detected over a 24-h monitoring period, kindling stimulation was terminated and LFP-video monitoring was continued for up to 4 days to assess daily incidences of spontaneous seizures. Spontaneous seizures were recognized by discharges in LFP recordings and accompanying motor seizures [17,18,39].

During baseline monitoring, no spontaneous seizures were detected in any of the mice examined, except for four events of spontaneous discharges recorded in one female mouse. These “baseline” discharges were observed during a 10-h period but not in the subsequent 30 h of monitoring and were not accompanied by evident convulsive behavior. The morphologies of these “baseline” discharges featured repetitive poly-spikes in the hippocampus and incremental rhythmic events in the piriform cortex. The hippocampal discharges had an earlier onset and longer durations relative to the piriform discharges (Supplementary Fig. 2). These features were distinct from those of spontaneous discharges observed in kindled mice [18]; see also below). A previous study has examined spontaneous seizures in a mouse model (male C57 black mice) of pilocarpine-induced epilepsy [40]. Continuous LFP-video monitoring revealed no spontaneous seizures after electrode implantation surgery, but LFP abnormalities such as sporadic synchronous bursts were observed in two mice. The “baseline” discharges we observed from the female mouse and the sporadic synchronous bursts observed from male C57 mice [40] might result from complications of intracranial electrode implantation (see Ref. [41] for more relevant information). While this remains a topic of further investigation, we considered these “baseline” discharges as temporary hyper-excitable events as hippocampal AD thresholds and durations, the number of hippocampal stimuli needed to reach the kindled state and total seizure burden obtained from this mouse were comparable to those collected from other female mice. Although such “baseline” spontaneous discharges were not observed in male mice in our previous studies [17,18,21,27], further experiments that assess a larger cohort of female mice are needed to verify our present observations.

Spontaneous seizures were observed in female and male mice following different numbers of hippocampal kindling stimulations. Among the 10 female mice monitored following 50 stimulations, two displayed spontaneous seizures, including the one in which baseline discharges were detected. Of the 10 female mice evaluated, 7 received ≥80 stimulations, and 3 were euthanized after ≤68 stimulations owing to the loss of implanted electrodes or severe skin infection/lesion. Among the 7 female mice receiving additional kindling stimulation, spontaneous seizures of 5–8 events were observed in 4 of them following 80 (*n* = 2), 100 (*n* = 1), or 120 (*n* = 1) stimulations. Three of these mice in which spontaneous seizures were not detected after 120 stimulations (Table 1) died “spontaneously” or were euthanized owing to the above-mentioned complications after receiving an additional 3–7 stimulations. In contrast, no spontaneous seizure was detected from male mice following 50 stimulations, and only one spontaneous seizure was observed from one male mouse following 80 stimulations. Among 11 of the 12 male mice that underwent further kindling, spontaneous seizures comprising 8–25 events were observed in 7 and 4 mice following 100 and 120 stimulations, respectively (Table 1). The percentages of animals in which spontaneous seizures were detected following ≤80 stimulations were significantly greater among female mice than among male mice (Chi-square test, *x*^2^ = 20.06, *z* = 4.478, two-tailed *p* < 0.0001 after 50 stimulations; *x*^*2*^ = 12.23, ^z^ = 3.497, two-tailed *p* = 0.0005 after 80 stimulations; Table 1). However, although the sample sizes were small, our data showed that female mice seemed to have a lower or more variable tendency to experience spontaneous seizures following ≥100 stimulations compared to male mice (Table 1).

**Table 1:** Measurements of spontaneous seizures.

Numbers of hippocampal stimulations	Times of LFP-video monitoring (hours)		Numbers and percentiles of mice with detected spontaneous seizures	
	females	Males	females	males
50	21.5 ± 1.5	20.8 ± 1.0	2 of 10, 20 % ∗	0 of 12, 0 %
80	23.4 ± 2.03	25.4 ± 2.4	2 of 7, 28 % ∗	1 of 11, 8 %
≥100	52.2 ± 15.25	50.6 ± 6.19	1 of 5, 20 % 100 stimulations	7 of 11, 63 % 100 stimulations
			1 of 4, 25 % 120 stimulations	4 of 4, 100 % 120 stimulations

Continuous LFP-video monitoring was performed to detect spontaneous seizure. The monitoring times after 50 and 80 hippocampal stimulations referred to those in the first session conducted within 36 h following the 50th or 80th stimulation. For female mice, the total monitoring times after 80 stimulations were 44.6 ± 10.66 h as some mice underwent further monitoring after the first session. The monitoring times after ≥100 stimulations denoted those in a monitoring session of up to 4 consecutive days, and data were pooled according to sex because of limited observations in female mice. The LFP-video monitoring times listed in the 2nd main column were not significantly different between female and male mice (Mann–Whitney *U* test; 50 stimulations, *u* = 70, *z* = 0.63, *p* = 0.526; 80 stimulations, *u* = 46, *z* = 0.30, *p* = 0.764; >100 stimulations, *u* = 70, *z* = −0.084, *p* = 0.933). Incidences (percentiles) of spontaneous seizures detected following 50 and 80 stimulations were greater in female than in male mice (∗, Fisher’s exact test, *p* < 0.0001 after 50 stimulations; *p* = 0.0005 after 80 stimulations). Seizure incidences after 100 or 120 stimulations were not statistically compared between females and males due to small sample sizes. Overall, the rate of successful extended kindling was lower in female mice than in male mice owing to the loss/malfunction of implanted electrodes or health-related complications, which compromised the assessments of sex-related differences in spontaneous seizures.

**Fig. 4:**
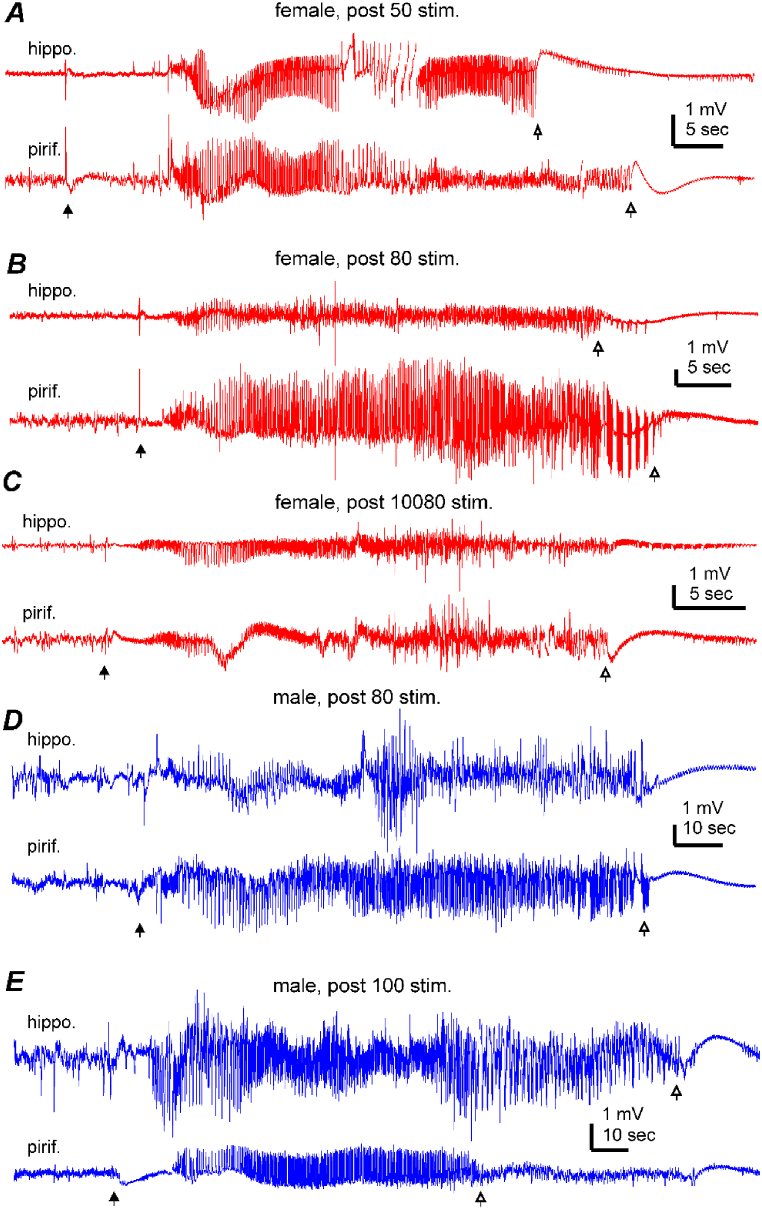
Spontaneous discharges observed following extended hippocampal kindling. **A-C**, LFP epochs were collected from 3 female mice (red traces in **A**-**C**) and 2 male mice (blue traces in **D**-**E**) at indicated experimental time points. Hippocampal (hippo.) and piriform cortical (pirif.) signals were monitored simultaneously in individual mice. Putative discharge onset and termination indicated by filled and open arrows. Discharges in A, B, D and E were associated with stage 3 motor seizures (supplementary videos 3, 4, 6, and 7). Discharges in C associated with a stage 5 motor seizure (supplementary video 5).

**Fig. 5:**
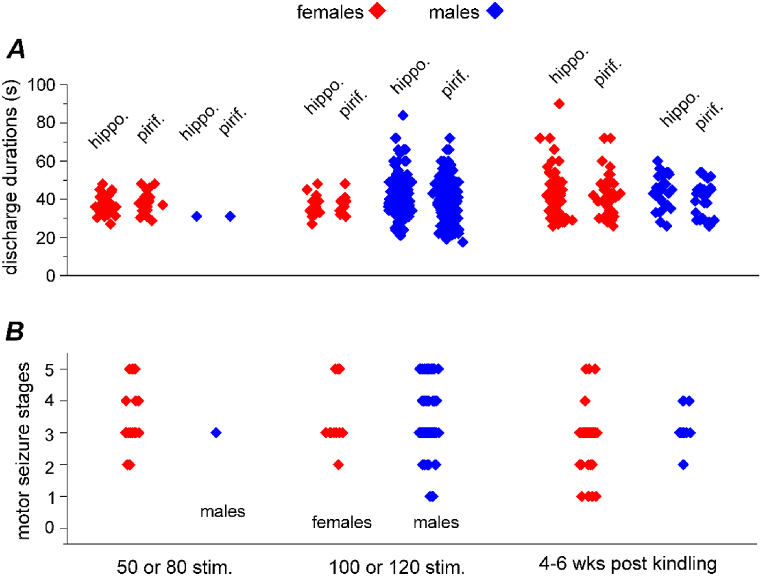
Measures of spontaneous seizures. The durations of spontaneous discharges (**A**) and stages of corresponding motor seizures (**B**) were measured following 50 or 80 stimulations (3 female mice and 1 male mouse), 100 or 120 stimulations (2 female and 11 male mice) and 4–6 weeks after termination of kindling (3 female and 4 male mice). Discharge measures from hippocampal (hippo.) and piriform (pirif) cortical areas are indicated. No significant sex-related differences were detected in these measures (Kruskal-Wallis test, Chi-square = 23.136, *df* = 11, *p* ≥ 0.237).

Examples of spontaneous seizures observed in female or male mice following 50, 80, or 100 stimulations are presented in Fig. 4A–E and Supplementary videos 3–7. Overall, all the spontaneous discharges co-occurred in the stimulated hippocampal and unstimulated piriform/cortical areas, and corresponding regional discharges began concurrently with low voltage signals and then continued with similar repetitive spike waveforms (Fig. 4A-E). Motor seizures corresponding to these discharges were mainly of stages 3–5 and featured forelimb clonus, rearing, and falling. Similar spontaneous seizures were observed in two female mice and three male mice 4–6 weeks after the termination of kindling stimulation. The measurements of spontaneous discharge durations and motor seizure stages obtained from female and male mice are depicted in Fig. 5A and B. Data collected following 50 or 80 stimulations, 100 or 120 stimulations, and 4–6 weeks after termination of kindling were pooled to generate relatively large sample sizes for statistical comparisons. No significant sex-related differences were detected in these measures (Kruskal-Wallis test, Chi-square = 23.136, *df* = 11, *p* ≥ 0.237), suggesting that spontaneous seizures occurring in female and male mice display largely comparable electrographic and convulsive features.

We also explored whether female and male mice differed in cluster expression of spontaneous seizures. Seizure clusters were defined as ≥4 consecutive seizures with inter-seizure duration of ≤120 min according to our previous examinations in kindled male mice [42]. Based on this definition, there were 14 clusters in 233 consecutive spontaneous seizures observed from male mice, whereas no cluster was found in 53 consecutive spontaneous seizures detected in female mice. However, these results are inconclusive regarding sex differences in seizure clusters because the numbers of observed consecutive spontaneous seizures, particularly those in the female group, are relatively small and limited for appropriate statistical evaluation.

#### 3.1.4 Hippocampal interictal spikes (ISs) and IS associated fast ripples

**Fig. 6:**
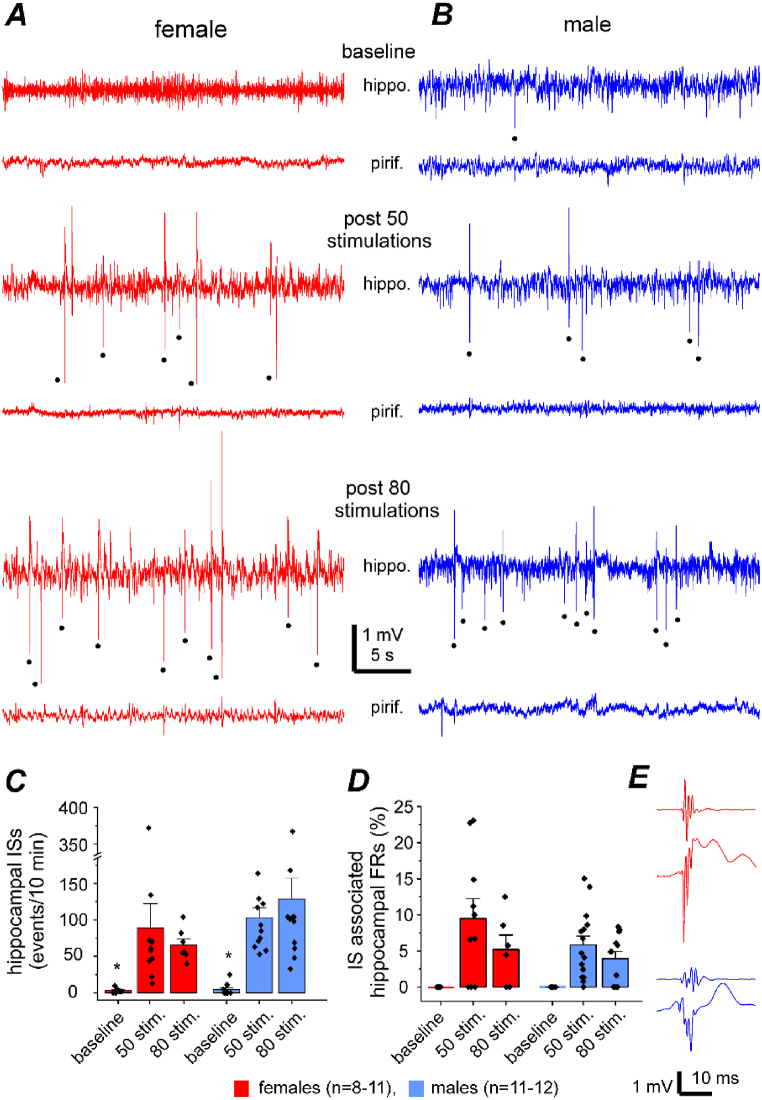
Measures of hippocampal interictal spikes (ISs). **A**-**B**, epochs of local field potentials (LFPs) collected from 2 mice before kindling (baseline) and following 50 or 80 hippocampal stimulations (stim.). LFPs were recorded simultaneously from hippocampal (hippo.) and piriform cortical (pirif.) areas. Detected baseline spikes and hippocampal ISs denoted by filled circles. **C**, rates of hippocampal baseline spikes and ISs were measured from 10-min data segments with dominant delta activity. **D**, rates (%) of fast ripples (FRs) that were associated with hippocampal ISs. Means and SE with individual data points presented in C-D. Sex-related differences were not significant in these measures, but rates of hippocampal ISs were significantly greater than those of baseline hippocampal spikes (Kruskal-Wallis test, Chi-square = 46.05, df = 5, p < 0.0001; ∗, baseline vs. 50 or 80 stimulations, Dunn’s multiple comparisons test, p ≤ 0.025). **E**, examples of IS-associated hippocampal FRs recorded from a female (red) or male (blue) mouse. Bottom traces present original LFP signals that were recorded in a frequency band of 0.1–1000 Hz. Top traces illustrate corresponding signals after treatment with a band passing filter of 250–500 Hz.

Spontaneous ISs were consistently observed in various animal models of epilepsy [43,44], including the extended kindling model [17,19,[45], [46], [47], [48], [49]]. Variations of IS frequencies with estrous cycle states have been demonstrated in rat models of kainic acid or pilocarpine induced epilepsy [50]. We therefore explored sex differences in expression of hippocampal ISs in our model. As kindled animals exhibited spontaneous ISs predominantly during inactive behaviors (such as sleep and immobility), we assessed hippocampal ISs in data segments that were collected while mice were asleep or in stable immobility. ISs were recognized as intermittent spike waveforms that displayed amplitudes of ≥6 standard deviations relative to the mean background signals and durations of ≤200 ms. Evident hippocampal ISs were observed in female and male mice following kindling (Fig. 6A and B). The rates of hippocampal ISs were 57.9 ± 11.6 and 103.2 ± 13.58 events/10 min following 50 stimulations and 65.7 ± 8.14 and 128.6 ± 28.92 events/10 min following 80 stimulations in female and male mice, respectively (Fig. 6C). There was no significant sex-related difference in these measures, but IS rates were significantly greater than the rates of baseline hippocampal spikes in both female and male mice (3.45 ± 0.99 and 4.67 ± 2.20 events/10 min; Kruskal–Wallis test, Chi-square = 46.05, *df* = 5, *p* < 0.0001; Dunn’s multiple comparisons test, p ≤ 0.025, baseline vs. 50 or 80 stimulations; Fig. 6C).

Fast ripples (FRs) with oscillatory activities of 250–500 Hz are considered an electrographic biomarker of epilepsy [43,44]. To assess FRs that were associated with hippocampal ISs, original IS signals were treated with a band-passing filter (250–500 Hz) for FR detection, and detected FRs were presented as percentiles of total hippocampal ISs (Fig. 6D and E). The FR rates estimated for female and male mice were 9.5 ± 2.7 % and 5.9 ± 1.21 % following 50 stimulations and 5.2 ± 2.0 % and 3.9 ± 0.98 % following 80 stimulations, respectively (Fig. 6D). Sex-related differences in these FR measures were not significant (Kruskal–Wallis test, Chi-square = 2.8322, *df* = 3, *p* ≥ 0.418).

#### 3.1.5 Hippocampal theta rhythms

**Fig. 7:**
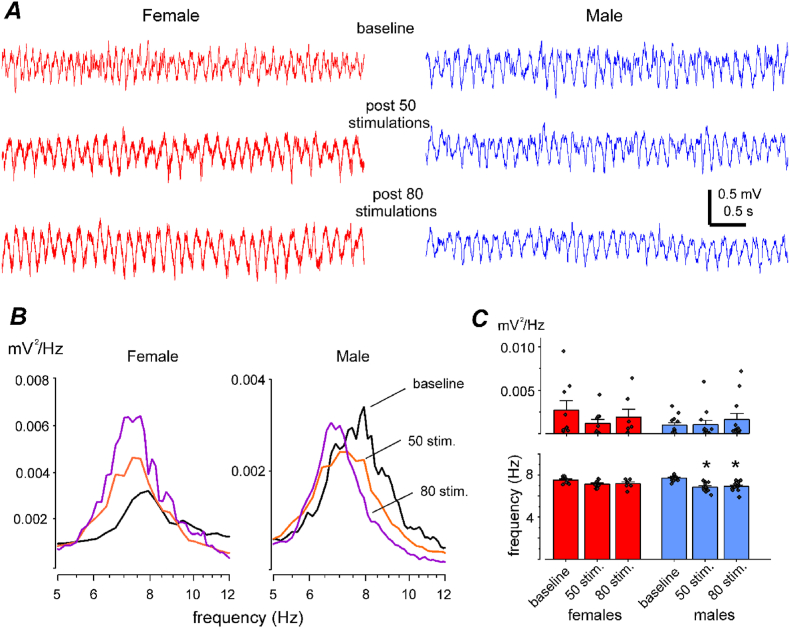
Changes in hippocampal theta rhythm following kindling. **A**, examples of hippocampal theta rhythm sampled from two mice before kindling (baseline) and following 50 or 80 hippocampal stimulations. **B**, corresponding spectral plots. Each plot was generated from 4 to 6 data segments including illustrated signals and averaged for presentation. Black lines denote baseline plots. **C**, measurements of theta peak frequencies and corresponding powers. Data (mean ± SE with individual data points) obtained from 7 to 10 female mice and 12 male mice. Theta frequencies following 50 and 80 stimulations were significantly decreased from baseline in male mice (∗, one-way ANOVA, (*f* = 9.311, *p* < 0.0001; Tukey’s post-hoc test, *p* < 0.001).

The rodent hippocampus exhibits theta rhythms (5–12 Hz) during active behaviors such as exploration and movement as well as in alert immobility, and hippocampal theta rhythms are thought to be important for memory-related information process [51,52]. Decreases in hippocampal theta rhythms have been observed in rodent models of kainic acid, pilocarpine or stroke-induced epilepsy [[53], [54], [55]] as well as in male mice following extended hippocampal kindling [19]. Decreased theta rhythms may be relevant to seizure genesis because elevated theta rhythms are postulated to represent a seizure-resistant network activity state [[56], [57], [58], [59]]. However, except for a recent study that demonstrated the existence of sex-related differences in theta rhythm alterations in a rat model of early-life seizures [60], the influence of sex on the changes in hippocampal theta rhythm remain largely unexamined in animal models of epilepsy. We therefore explored sex-related differences in alterations of hippocampal theta rhythms in kindled mice. Examples of hippocampal theta rhythms and corresponding spectral plots obtained from a female mouse and a male mouse are presented in Fig. 7A and B. Overall, the mean frequencies of theta rhythm were decreased in kindled female and male mice relative to baseline levels (7.5–7.1 or 7.2 Hz for females and 7.7 to 6.9 Hz for males) (Fig. 7C). One-way ANOVA revealed a significant difference in the effect of sex on kindling-induced decreases in theta frequencies (f = 9.311, p < 0.0001). The decreases were significant in the male group (Tukey’s post-hoc test, 50 or 80 stimulations vs. baseline, p < 0.001), but not in the female group (p ≥ 0.196). There was no significant effect of sex on changes in theta powers (Kruskal-Wallis test, Chi-square = 4.50283, df = 5, p = 0.47951).

## 4 Discussion

In this study, we examined the influence of sex on the genesis of hippocampal kindling-induced seizures in middle-aged C57 black mice. Specifically, we aimed to explore whether female and male mice differ in kindling epileptogenic process and in genesis of spontaneous epileptic seizures following extended kindling. Our results showed that there were sex-related differences in the development and severity of evoked seizures, in the manifestation of spontaneous seizures following extended kindling, and in the changes in hippocampal theta rhythm.

### 4.1 Sex-related differences in kindling epileptogenic process

Electrical kindling is a widely used model of TLE [61]. In the kindling model, the progression and severity of evoked seizures can be reliably measured in individual animals hence providing information about kindling epileptogenic process. Sex-specific differences in kindling-evoked seizures have been previously examined. For example, in response to amygdala kindling, female and male rats showed similar seizure development as measured by the severity and duration of evoked motor seizures and AD [62,63]; nevertheless, there was a more evident drop in the AD threshold among female rats than among male rats [62]. The durations, frequencies, and amplitudes of the evoked AD and the stages of motor seizures were comparable between female and male rats following cortical kindling; however, there were sex-related differences in the extent of changes in the dendritic structures of cortical pyramidal cells [64]. With respect to kindling seizures in C57 black mice, hippocampal kindling induced more rapid progression of evoked seizures and longer hippocampal AD in male mice than in female mice [13]; corneal kindling evoked similar seizures in female and male mice [65] but the thresholds of corneal electroconvulsive stimulations or corneal kindling were lower in female mice than in male mice [[14], [15], [16]]. Chemical kindling particularly *via* repeated applications of pentylenetetrazol (a GABA_A_ receptor antagonist) is also used to assess epileptogenic process in rodent models [66]. The severity of pentylenetetrazol kindling seizures was greater in male rats than in female rats [67] or appeared to be similar between female and male rats [68,69]. Differences in multiple experimental factors, including animal species, electrical kindling sites and kindling approaches (chemical *vs.* electrical), may explain, at least partly, the variable sex-related differences observed in these studies [5].

We found that relative to male mice, female mice required fewer hippocampal stimulations and shorter cumulative AD to reach the kindled state (three consecutively evoked stage 5 motor seizures) and exhibited greater motor seizure severity. These sex-related differences were unlikely related to kindling stimulation strengths as hippocampal stimulations were applied to individual mice at 25 % above their initial AD thresholds and the sex differences in AD threshold measures were relatively small. Together, these results indicated that middle-aged female mice are more susceptible than male mice to evoked seizures in the hippocampal kindling model. The main outcomes are dissimilar between our present experiments and the previous study [13]. Differences in the ages of the animals may have been a major contributing factor to the faster seizure development observed in adult male mice in the previous study [13] compared with that seen in the middle-aged female mice in our present experiments given that similar hippocampal kindling was conducted in C57 black mice in both studies. However, further work is needed to verify this age-dependent difference and to investigate underlying mechanisms (see below).

### 4.2 Sex-related differences in the manifestation of spontaneous epileptic seizures

Epilepsy is defined by reoccurrence of unproved or spontaneous seizures [1]. It is therefore important to investigate the influence of sex on the genesis of spontaneous epileptic seizures in animal models. Several studies have examined the development and incidence of spontaneous epileptic seizures in C57 black mice. For example, relative to male mice, adult female mice showed earlier onset or higher rates of spontaneous epileptic discharges following the intra-hippocampal injection of kainic acid [9,10]. Aging (19–20 months old) female mice experienced more severe motor seizures than male mice following the intra-nasal application of kainic acid, whereas, under the same conditions, similar seizure activities were detected in both female and male adult mice [8]. Similarly, adult female and male mice displayed comparable seizure activities following the intra-peritoneal injection of pilocarpine [13]. Variable sex-related differences in spontaneous seizures have bee observed in kainic acid or pilocarpine rat models [5,70,71]. As the development of spontaneous seizures in these models is associated with brain injuries of varying degrees, the sex-dependent differences in spontaneous seizure outcomes may be due to the extent of brain injury, at least partly. Unlike the kainic acid and pilocarpine models of epilepsy, the development of spontaneous seizures following extended kindling is not associated with gross brain injury [46, 49, [72], [73], [74]]. Accordingly, our extended kindling model allows the genesis of spontaneous seizures relatively independent of gross brain injury [18], thereby complementing other models and enabling a comprehensive understanding of the influence of sex on the genesis of spontaneous epileptic seizures.

We explored in the present experiments whether middle-aged female and male mice differ in genesis of spontaneous seizures following extended hippocampal kindling. In all male mice examined in the present experiments, except for one event detected following 80 stimulations, all spontaneous seizures were observed following 100 or 120 stimulations. In contrast, spontaneous seizures were detected in 2 out of 10 female mice following 50 stimulations and in 2 out of 7 female mice following 80 stimulations. The proportion of female mice in which spontaneous seizures were detected following ≤80 stimulations was greater than that of male mice. Female mice seemed to show an opposite trend in response to further kindling, as spontaneous seizures were only observed in 2 out of 4 mice following 100 stimulations and in only one of the four mice following 120 stimulations. While the propensity to exhibit spontaneous seizures following extended kindling appeared to be more variable in female mice than in male mice, with some female mice being prone and others resistant to seizure genesis, our present data are inconclusive because observations of spontaneous seizures in different stages of extended kindling were obtained from a small cohort of mice particularly in the female group. While sex-related differences in genesis of spontaneous seizures following extended kindling remain to be further examined in our model, the experimental protocol used in our present experiments may be applicable in future studies to assess spontaneous seizures in a larger cohort of female and male mice.

### 4.3 Sex-related differences in the expression of hippocampal ISs and theta rhythm alterations

We found that both female and male mice expressed evident hippocampal ISs following kindling and that the mean rates of hippocampal ISs and IS-associated FRs were not significantly different between female and male mice. The peak frequencies of hippocampal theta rhythm were significantly decreased from baseline in male mice, whereas kindling-induced changes in theta peak frequencies were not significantly different from baseline in female mice. These findings were unexpected given that the proportion of female mice in which spontaneous seizures were detected following ≤80 stimulations was greater than that of male mice (see above). However, as our present observations were obtained from a relatively small number of mice and estrous cycle states were not assessed in our experiments (see below), further work is needed to verify the sex-related differences in expression of ISs and changes of hippocampal theta rhythms in our model.

### 4.4 Limitations of our present study

There are several weaknesses/limitations in our present study. A major weakness of our present experiments is that hippocampal kindling was conducted in a relatively small cohort of mice. In particular, the rate of successful extended kindling was lower in female mice than in male mice owing to the loss/malfunction of implanted electrodes or health-related complications, which compromised the assessments of sex-related differences in spontaneous seizures, hippocampal ISs, and changes in theta rhythm in the late phase of extended kindling.

Another major weakness is that we did not assess estrous cycle states and sex hormone levels in middle-aged mice. Sex differences in biological aging are thought to be strongly related to sex hormones because circulating levels of sex hormones decline substantially in aging females but only moderately in age-matched males. Most women approaching 50 years of age undergo perimenopause, a transition state characterized by the disruption of multiple sex hormone-regulated neurological systems [75]. Such disturbances can increase the risk of developing neurological disorders, including epilepsy [76]. For C57 black mice, females have peak levels of ovarian hormones by 3–6 months of age and approach the endocrine equivalent of human perimenopause/menopause by 9–12 and 13–16 months of age respectively [77, 78]. In addition, approximately 60–70 % of the aging mice spontaneously transition into a state of constant estrus characterized by sustained levels of plasma 17β-estradiol and low levels of progesterone that can last 10–100 days [79]. By contrast, serum testosterone levels in male C57 black mice remain relatively stable by 8 months of age and declined to 60 % of adult level at 24 months of age [80]. Sex hormones are pivotal for brain functions [81]. Abundant studies have demonstrated profound effects of sex hormones on hippocampal synaptic and ionic activities [82,83]. Sex hormones and estrous cycle status are known to affect seizure susceptibility [84,85]. In general, progesterone is considered to have anticonvulsant actions, and estrogen and androgens have both proconvulsant and anticonvulsant effects depending on experimental conditions (such as hormone levels, animal models and seizure types). Seizures are also known to affect sex hormone levels and reproductive functions. For example, amygdala kindling seizures in male rats resulted in an increase in serum testosterone, estradiol, and prolactin, accompanied by a significant increase in testis, epididymis, and pituitary weight [86]. When examined in female rats, amygdala kindling seizures arrested ovarian cyclicity and raised serum estradiol levels, together with increased pituitary weight and polyfollicular ovaries [87]. We hypothesize that changes in estrous cycle states and decreases in ovarian hormones particularly progesterone as well as related alterations at brain cellular and circuit levels in middle-aged/aging female mice are important factors attributing to seizure susceptibility. Future works are needed to test this hypothesis. Specifically, assessments of estrous cycle states in individual mice during extended kindling and measurements of sex hormone levels in kindled mice with or without detectable spontaneous seizures may provide mechanistic information regarding sex influences on kindling seizure outcomes in our model.

An additional area for improvement is to conduct continuous LFP-video monitoring more frequently during kindling process and to detect spontaneous seizures and related alterations more accurately in individual mice. Despite these weaknesses and limitations, it is our hopes that our present observations may provide a framework for further in-depth investigations on and contribute to the understanding of the influence of sex on new-onset epilepsy in middle-aged and older patients.

## CRediT authorship contribution statement

**Hongmei Song:** Methodology, Data curation, Conceptualization. **Yapeng Liu:** Methodology, Data curation, Conceptualization. **Yuqing Sun:** Data curation. **Bryan Mah:** Data curation. **Yang Bai:** Funding acquisition, Conceptualization. **Liang Zhang:** Writing – review & editing, Writing – original draft, Supervision, Funding acquisition, Data curation, Conceptualization.

## Ethics statement

All experimentations were reviewed and approved by the Animal Care Committee of University Health Network (Animal User Protocol 986.40) according to the Guidelines of the Canadian Council on Animal Care.

## Data availability statement

All data presented in this study will be available in the data repository “GIN Modern Research Data Management for Neuroscience”. The DOI for the dataset is: https://doi.org/10.12751/g-node.f7bbgm.

## Funding

This work has been funded by a grant from the Epilepsy Research Program of 10.13039/100008914Ontario Brain Institute and supported by Program of Provincial Science and Technology Development of Jilin, China (No. 20210402008GH).

## Declaration of competing interest

The authors declare that they have no known competing financial interests or personal relationships that could have appeared to influence the work reported in this paper.

## Figures and Tables

**figs1:**
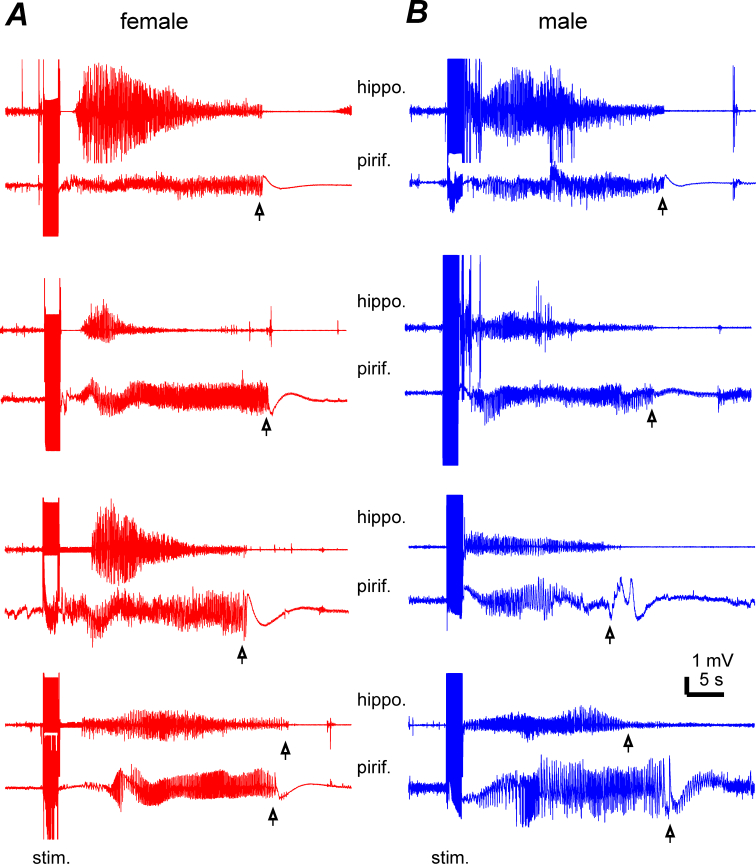


**figs2:**